# Community stakeholders’ perspectives on supporting the well-being of older adults: a focus group study on lessons learned during the COVID-19 pandemic

**DOI:** 10.1186/s12889-026-27862-8

**Published:** 2026-05-22

**Authors:** Djoeke Besselink, F. D. Schaap, H. Jager-Wittenaar, E. J. Finnema, F. van der Lucht

**Affiliations:** 1 Health Science-Nursing Science and Education, University of Groningen, University Medical Center Groningen, Groningen, 9713 GZ the Netherlands; 2 Research Group Healthy Ageing, Allied Health Care and Nursing, Hanze University of Applied Sciences, Petrus Driessenstraat 3, Groningen, 9714 CA the Netherlands; 3 FAITH research, Leeuwarden/Groningen, the Netherlands; 4 Regional Public Health Service, GGD Groningen, Hanzeplein 120, Groningen, 9713 GW the Netherlands; 5 Research Group Living, Wellbeing and Care for older people, NHL Stenden University of Applied Sciences, Leeuwarden, 8917 DD the Netherlands; 6 Faculty of Physical Education and Physiotherapy, Department of Physiotherapy, Human Physiology and Anatomy, Research Unit Experimental Anatomy, Vrije Universiteit Brussel, Brussels, 1090 Belgium; 7 Department of Gastroenterology and Hepatology, Dietetics, Radboud university medical center, Nijmegen, the Netherlands; 8 Executive Board, NHL Stenden University of Applied Sciences, Rengerslaan 8-10, P.O. Box 1080, Leeuwarden, 8900 CB the Netherlands; 9 Centre for Prevention, Lifestyle and Health, National Institute of Public Health and the Environment, Bilthoven, 3721 MA the Netherlands

**Keywords:** Community-based care, Older adults, Well-being, Focus group study, Constrained health and social care systems, COVID-19 pandemic

## Abstract

**Background:**

By 2050, one in four Dutch residents will be aged 65 years or older, intensifying the need to sustain older adults’ well-being amid growing pressure on health and social care systems. The COVID-19 pandemic served as a stress test, revealing both vulnerabilities and resilience in the support for older adults.

**Methods:**

We conducted a focus group study to examine the requirements for promoting the well-being of community-dwelling older adults in the context of increasingly constrained health and social care systems, as perceived by community care professionals, informal caregivers, older adults, and public health professionals during the COVID-19 pandemic. Six online, semi-structured focus groups with a total of 22 participants were held between October 2021 and May 2022 and analysed using abductive thematic analysis.

**Results:**

Three overarching themes emerged. Firstly, participants called for person-centred approaches that reflect the diverse realities of older adults, despite constrained resources in health and social care systems. Secondly, participants emphasised strategies extending beyond medical care, focusing on prevention and collaboration across sectors. Thirdly, participants stressed empowering older adults to maintain autonomy, resilience, and social connection through proactive outreach and community-based support.

**Conclusions:**

Promoting the well-being of older adults requires a continuous commitment to preventive, context-sensitive approaches, even amid constrained resources within health and social care systems. This entails embedding adaptivity within national frameworks to adapt to local contexts, fostering cross-sector collaboration to deliver person-centred care, and creating environments that empower older adults to actively prepare for ageing.

**Supplementary Information:**

The online version contains supplementary material available at 10.1186/s12889-026-27862-8.

## Background

By 2050, one in four Dutch residents will be aged 65 years or older, and many will experience multiple chronic conditions, increasing the demand for coordinated, long-term care [1, 2]. At the same time, the Dutch healthcare system faces critical workforce shortages, partly due to the ageing of the caregiver population [2, 3]. These demographic and systemic shifts place mounting pressure on the accessibility and quality of care for older adults [3, 4].

In response, Dutch (public) health strategies reflect a move away from reactive, disease-focused care to community-based care supporting older adults to live at home for as long as possible [5–7]. In the Dutch context, community-based care can be understood as the operational interface where primary care and public health intersect and are jointly enacted in practice [8]. Primary care includes a range of health and social care services delivered outside institutional settings by district nurses, domestic helpers, social workers, and local welfare organisations, alongside the care provided by informal caregivers [8, 9]. The Dutch public health model operates at the municipal and regional level and emphasise prevention and health promotion [10]. While community-based services are intended to play a bridging role, linking individual care trajectories with broader preventive and social policy goals, this remains challenging in practice due to staff shortages and fragmentation across sectors and governance levels [9, 10]. This highlights the need for adaptive, locally anchored strategies that better align primary care and population health functions within constrained resource environments [9–13].

The COVID-19 pandemic served as a real-world stress test, exposing both the vulnerabilities and resilience of community-based support structures. As healthcare capacity was redirected toward treating COVID-19 patients, routine and preventive services were postponed or cancelled [14]. At the same time, social distancing measures and lockdowns significantly limited in-person interactions, disrupted home visits, and restricted mobility across service areas. These disruptions undermined the continuity, responsiveness, and relational foundations of care for older adults, particularly those who were already frail or those with chronic conditions [14–16]. Consequently, studies suggest that the pandemic adversely affected the well-being of older adults, increasing their risk of loneliness, functional decline, and reduced quality of life [17–19].

At the same time, the crisis revealed creative and resilient responses with lessons for future care delivery [15, 20, 21]. For example, there was an accelerated adoption of digital health tools, including telehealth consultations and remote monitoring, which emerged as viable alternatives to in-person care and helped maintain contact with older adults when face-to-face services were limited [22]. Furthermore, at the beginning of the pandemic, a rise in grassroots initiatives and informal support networks was seen in communities, where neighbours, volunteers, and informal caregivers stepped in to deliver groceries, provide social check-ins, or help bridge the digital divide for older adults [23]. Such community ties can provide vital emotional and instrumental support sustaining older adults’ well-being [24].

Gaining insights into the experiences and responses of stakeholders in community-based care during times of crisis can provide critical insights into how community-based care can be strengthened in the face of both acute emergencies and long-term systemic constraints. Therefore, this study examined the requirements for supporting the well-being of community-dwelling older adults in contexts of constrained resources within health and social care systems, as perceived by community care professionals, informal caregivers, older adults, and public health professionals during the COVID-19 pandemic. In doing so, the study contributes to the development of resilient, community-oriented care systems capable of sustaining older adults’ well-being amid both temporary shocks and long-term demographic and structural challenges.

## Methods

### Study design

This qualitative focus group study was conducted to answer the research question ‘What are the requirements for supporting community-dwelling older adults in the context of constrained resources within health and social care systems, according to insights from community care professionals, informal caregivers, older adults, and public health professionals during the COVID-19 pandemic?’. Focus groups were considered a feasible methodology to capture the diversity of perspectives of the various stakeholders within community-based care for older adults and to discover shared experiences and perceptions among participants. The theoretical framework underpinning our study was abductive reasoning. Abductive reasoning involves exploring a phenomenon through an iterative process, applying both theoretical and empirical perspectives to arrive at the “best explanation” or solution [25]. In other words, it enables us to generate new insights by creatively integrating existing knowledge with emerging data. This approach was chosen because it supports a rigorous, transparent synthesis of data and theory, allowing for nuanced explanations and actionable recommendations that are theoretically grounded and responsive to the complexity of the pandemic context [26, 27].

To guide the theoretical understanding of how older adults can be supported, this study drew on the public-health framework for healthy ageing [5]. This framework holds that healthy ageing is influenced by a wide range of interrelated factors (e.g., biological, behavioural, social, and environmental) that interact across the life course. It emphasises the importance of creating supportive environments, reducing health inequalities, and promoting functional ability to enable well-being in older age [5].

### Study sample and recruitment

Participants were recruited through an open call within the FAITH research community. FAITH research is a Dutch network of universities of applied sciences and healthcare organisations in the Northern Netherlands focused on frailty prevention in older adults. Eligible participants included community care professionals and policymakers from health and social care organisations, informal caregivers, and older adults’ representatives that were proficient in Dutch. This last group consisted of older adults who were part of a seniors’ organisation. They were included to share insights into the experiences of the members of their organisations during the pandemic. To reach underrepresented groups, such as informal caregivers and older adults’ representatives, we employed purposive and snowball sampling through referrals from initial participants.

All participants were required to be based in the Northern Netherlands during the pandemic. The Northern Netherlands is a predominant rural area that constitutes of the provinces Groningen, Friesland and Drenthe. Like other rural European regions, Northern Netherlands is undergoing demographic decline, with a shrinking population and a growing share of older adults, while simultaneously facing ongoing shortages in health and social care resources [28]. This combination intensifies pressure on both formal and informal care provision [2]. Therefore, we considered Northern Netherland a setting relevant to challenges in sustaining the support for older adults while facing short- and long-term systemic constraints.

In total, six focus groups, each compromising 3–6 participants, were conducted to foster rich and dynamic discussions in which participants could build on one another’s insights [29]. The focus groups were held between October 2021 and May 2022. Following a strict lockdown from October 2021 to January 2022, COVID-19 measures were gradually eased towards May 2022 [30]. This period of fluctuating COVID-19 measures provided a valuable opportunity to examine participants’ experiences across different phases of the pandemic. Although we initially aimed to group participants homogeneously to encourage meaningful discussions among individuals with similar experiences and responsibilities [31], practical scheduling constraints made this difficult to achieve fully. As a result, two focus groups included a mix of participants with different backgrounds and roles (see Results).

### Topic list

A semi-structured topic list was developed for this study, guiding the focus groups. This topic list was informed by key principles of value-based health care, which focus on delivering care that improves outcomes important to older adults, while ensuring efficient use of resources [32]. Supplementary material 1 provides a translated version of the topic list. The topic list focused on changes in the way older adults’ well-being was supported during the pandemic, barriers experienced by participants to meet the needs of older adults to maintain their well-being and potential solutions to support older adults while opportunities for community-based service delivery were scarce.

### Procedure

Before the focus groups, participants signed an informed consent form. All focus group sessions lasted 60 min and were conducted online via Microsoft Teams, due to COVID-19 restrictions. Besides, professionals were stationed in various provinces and, therefore, unable to come to one physical location.

Focus groups were facilitated by a moderator and an assistant, both of whom had extensive experience in qualitative methods. The roles of moderator and assistant were filled in iteratively by the principal investigator (DB) and the second author (FS). The moderator guided the conversations. The assistant facilitated technically, helped participants if any technical difficulties occurred, took notes, managed audio recordings, probed if needed, and ensured that all aspects of the interview guides were covered.

The focus group began with an introduction to the researchers and participants, along with an explanation of the purpose of the study. Then, the moderator asked participants to open a digital whiteboard called Mural. We chose to work with Mural as a tool to encourage more active engagement and reflection before the group discussion, ensuring that all voices were captured, including those who might be less vocal during open discussion. By first allowing participants to individually contribute and structure their thoughts in a shared digital space, all voices were initially captured in a more equalised format, reducing the immediate influence of hierarchical or professional status on verbal contributions. Once participants had completed their input in Mural, contributions were reviewed and discussed collectively, guided by the moderator. During this discussion, the moderator facilitated interaction by being attentive to differences in perspectives, inviting participants to elaborate on their views, and encouraging dialogue around contrasting contributions. This way, the subsequent group discussion could focus more efficiently on clarification, comparison, and deeper exploration of participants’ input.

All focus group sessions were audio recorded and transcribed by a professional transcription service in the original language. To maintain confidentiality, files were shared through an encrypted transfer system, stored on password-protected services and deleted by the transcription agency within 30 days after completion. Then, transcripts were formatted for analysis by removing any personal information of participants and details about specific facilities, colleagues, or institutions. All materials were securely stored in the institute’s digital research environment and will be retained for a minimum of 15 years. Translations into English were carried out during the final stage of manuscript preparation and subsequently reviewed by a native English speaker.

### Ethics

The study received ethical approval from the Hanze Ethical Review Board (HEAC 2021.009) and was conducted in accordance with the principles of the Declaration of Helsinki. Prior to participating in the focus groups, all individuals received an information letter outlining the study’s purpose and procedures. In addition, all participants provided written informed consent before the start of the study.

### Data analysis

Focus groups were analysed using an abductive thematic analysis approach, following the guidelines outlined by Thompson (2022) [27]. The entire analyses were done in the original language to ensure that interpretation remained grounded in participants’ working and contextual meaning.

In the first step, the principal investigator revisited the data by thoroughly reading and re-reading the transcripts, writing analytical notes and memos to capture initial impressions and points of ambiguity. Next, the principal investigator labelled raw data from two focus groups with open codes. The goal was to explore as much semantic meaning as possible to build a deeper level of comprehension of the data. This was done inductively, meaning that codes were generated from the data itself rather than being based on predefined categories or theoretical frameworks. Each code was provided with a label that described its meaning and when to use it, forming a preliminary codebook. The principal and second investigator collaboratively reviewed and refined this codebook. Then they independently applied the refined codebook to a third focus group, resolving discrepancies and finalising the codebook. The principal investigator used this codebook to code all transcripts.

Once all transcripts were coded, the principal investigator grouped the open codes into broader categories. The categories were then discussed by the principal and the second investigator. Here, a first connection between the empirical data and relevant theoretical perspectives was laid. The purpose was to move beyond descriptive coding and begin interpreting patterns in the data in light of existing concepts related to healthy ageing. For instances for which theories could not account for the data, these were treated as prompts for further interpretation. Meaning that this data was used as starting points to explore whether alternative explanations were needed to better account for the empirical findings.

In the final phase of analysis, themes were developed across all transcripts to identify commonalities and differences. All authors participated in a session to group and discuss how categories related to another and explained the phenomenon of supporting older adults in times of constrained resources. At this stage, relationships and patterns between themes were critically examined. Following the group discussion, the principal and second investigator further refined the themes and compared with existing theoretical knowledge. They selected the public health framework for healthy ageing because it integrates primary health care and public health perspectives to address the needs of older adults in relation to healthy ageing. This framework supported the clustering and interpretation of the emerging themes. For example, it emphasises not only strategies that reduce age-related losses, but also the importance of strengthening health systems that sustain capacity throughout ageing. Sustaining older adults’ capacity during the pandemic formed a central thread across the themes, and the framework helped organise them in a way that illuminated interactions across different levels of the health system. The final stage of analysis resulted in latent, conceptually rich themes that reflect the dynamic interplay between participants’ experiences during the pandemic and theoretical perspectives. Perspectives specific to different roles in supporting older adults (e.g., informal caregivers, policymakers, and community care professionals) are highlighted where relevant in the findings.

### Rigour, reflexivity and trustworthiness

In assessing the completeness of thematic development, sampling was informed by the study aims and pragmatic considerations of achieving adequate coverage across stakeholders’ perspective. During the final stages of data collection, we did observe a high degree of consistency in input by the focus groups. On this basis, the dataset was judged to provide sufficient depth and coverage to support the analytic claims.

Throughout data collection and analysis, the principal and second investigators engaged in biweekly reflexive sessions to iteratively interpret the data and examine how their professional backgrounds, theoretical knowledge and assumptions might shape the analysis. This process involved continual movement between individual excerpts and the dataset, fostering analytic depth and consistency. To enhance trustworthiness, preliminary interpretations were discussed in an interdisciplinary meeting with all authors, bringing together expertise to enable analytic triangulation and challenge potential bias. Reflexive memos and team discussions throughout the process strengthened the credibility and confirmability of the findings.

## Results

**Fig. 1:**
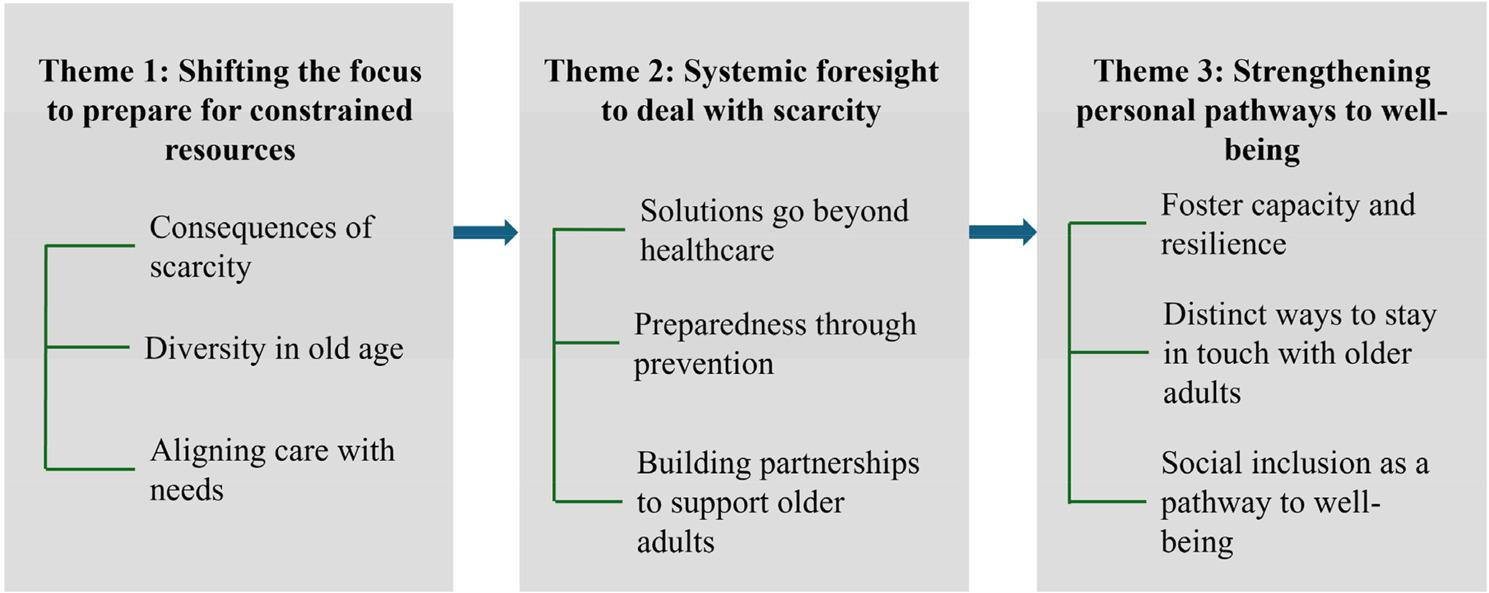
Overview of the overall findings of the study

We conducted six focus groups involving a total of 22 participants, with each focus groups compromising 3–5 participants. Table 1 provides an overview of participants and focus group compositions. Figure 1 shows an overview of the overall findings in our study.

**Table 1:** Participants and focus group compositions

Focus group	Participants	Description of (professional) backgrounds
1	5	Three community care professionals and two older adults
2	4	Community care professionals
3	4	Policymakers in (public) health
4	3	Policymakers in (public) health
5	3	Policymakers in (public) health
6	4	Informal caregivers and one older adult

*Notes.* Table 1 gives an overview of focus groups conducted, including the number of participants and their (professional) backgrounds

### Theme 1: shifting the focus to prepare for constrained resources

This theme reflects participants’ perspectives on the need to rethink support for community-dwelling older adults in a context of constrained resources during the pandemic, including staffing shortages, limited-service capacity, and rigid protocols. Participants emphasised the importance of flexible, responsive, and human-centred approaches that better reflect the lived realities of older adults, even when resources are constrained. This theme is organised into three subthemes: [1] Consequences of scarcity, which captures the impact of reduced resources during the pandemic; [2] Diversity in old age, which highlights the diversity in how older individuals responded to changes during the pandemic; and [3] Aligning care with needs, which emphasise the need for personalised and context-sensitive approaches to support older adults despite constrained resources.

#### Consequences of scarcity

Within all focus groups, participants discussed how prolonged reductions in care capacity during the pandemic gradually revealed critical gaps in the care for older adults. While the initial adjustments to service provision were generally accepted as necessary and temporary, informal caregivers and community care professionals noted that the prolonged duration of these limitations had a growing negative impact on the older adults they were caring for.

*“Well*,* it did strike me that right at the very start of it all*,* when everything was*,* well*,* still very unpredictable*,* that people found it easier to accept and were like “Okay*,* for the time being everything has to be organised differently.” And the longer it continued*,* the more it impacted people.”* Policymaker, focus group 3.

From the perspective of community care professionals, one key issue during the pandemic was the loss of routine in in-person contact with their clients, which they viewed as essential for identifying subtle changes in their overall health and well-being. They stressed that the absence of regular face-to-face interaction with their clients made it more difficult to detect early signs of decline, raising concerns about long-term health outcomes. Informal caregivers confirmed this, sharing first-hand experiences of health needs being missed or escalating unnoticed among older adults.

A common explanation for the decline in in-person contact mentioned across focus groups was that older adults themselves cancelled care visits, either due to fear of infection or because they did not want to be a burden. Community care professionals and informal caregivers noted that this resulted in some older adults choosing to manage independently, which was viewed with mixed feelings. On one hand, it was seen as a sign of independence, autonomy and self-reliance; on the other, as a potential risk for unmet needs among older adults and overburden of the informal caregivers during the pandemic.

*“I have heard people* [refers to clients] *say more often*,* “oh*,* well then we’ll just do it ourselves this once*,* because you have so much on your plate already”*,* or “then you have to come all suited up [refers to protective gear against infection] every time*,* so well*,* then I’ll just do it myself”. And while that may have a positive side*,* in the long run it worries you sometimes*,* because they might become overloaded.”* Community care professional, focus group 1.

#### Diversity in old age

A recurring theme across focus groups was the variation in how older individuals responded to changes during the pandemic. Community care professionals and informal caregivers drew on their direct experiences to illustrate how differences in personality, health status, and prior life experiences shaped individual responses. For example, community care professionals observed that some older adults adapted with resilience, while others struggled more visibly, particularly those with complex health needs or limited support networks.

*“I’ve experienced that often*,* with people with more complex problems*,* there might be more fear. I’m not sure if that is quite correct*,* but I had the impression that the more complex the situation was*,* the more people were also just*,* you know*,* in the grip of fear.”* Policymaker, focus group 4.

The policymakers considered diversity from a broader social-demographic perspective. They highlighted generational and socio-cultural influences, such as norms around self-sufficiency or reluctance to ask for help, as key to understanding how older adults navigated the crisis.

*“And there is often an uncertainty in taking action*,* and age*,* the age group*,* also plays a big part here. Especially the older generation of 90 plus*,* that whole*,* yes that whole pre-war…*,* that is really like: well*,* keep a stiff upper lip*,* eh.”* Policymaker, focus group 4.

A representative for older adults further expanded the understanding of diversity by challenging assumptions of vulnerability. Rather than viewing older adults as a homogenous group with declining capacity, they emphasised that many individuals in the so-called “third age” remain active, capable, and resilient, underscoring the need to avoid overgeneralisation in policy and practice.

“*And of course*,* not everyone is vulnerable*,* right*,* a lot of people in the third phase of life are vital*,* can still do a lot themselves.*” Older adult, focus group 1.

#### Aligning care with needs

Participants across all focus groups underscored the importance of tailoring care to the unique values, preferences, and life contexts of older adults. In the focus groups with community care professionals and policymakers, person-centred care was described as requiring not just service provision, but collaborative problem-solving, thinking with older adults to creatively adapt care under constrained conditions such as the pandemic.

*“I also think it is mainly about being able to think along with people [refers to clients]. Especially in home care*,* which just carried on*,* and they tried to brainstorm with people: ‘Well*,* how could you do this slightly differently’ or ‘What might help you?’ if clients didn’t see a way out. More focus should be placed on that.”* Policymaker, focus group 3.

Informal caregivers and representatives of older adults focused more on the relational and practical aspects to support older adults’ well-being, such as help with groceries, transportation, or maintaining social routines. These small, often informal acts were seen not just as practical support, but as critical for maintaining dignity and independence. Informal caregivers especially emphasised how simple, human interactions, like sharing a cup of coffee or playing music, could offer meaningful emotional support.

*“And it’s the small things actually*,* just giving a bit of attention in a creative way*,* a cup of coffee*,* a bowl of soup*,* a small music performance. And that lifts people out of that heavy*,* gloomy mood for a moment.”* Informal caregiver, focus group 6.

However, due to pandemic-related constraints, such as rigid protocols and staff shortages, community care professionals felt hindered in their ability to provide person-centred care. They explained that national policies, while well-intentioned, often failed to reflect the realities they encountered on the ground and constrained their ability to tailor support effectively. Together with policymakers, they advocated for greater local discretion and flexibility in care provision to be able to support older adults’ well-being and respond creatively to emerging needs during the crisis:

*“Well*,* what’s decided at a national level sometimes just doesn’t at all fit in with what’s still possible locally. That’s what I mean when I say municipalities should put the human touch first. If you can match national guidelines with local practice*,* it’s easier to put compassion first.”* Community care professional, focus group 2.

### Theme 2: systemic foresights to deal with scarcity

Theme 2 focuses on structural and systems-level strategies to support the well-being of older adults, despite constrained resources, with an emphasis on prevention, planning, and multisectoral collaboration. The theme describes a bigger-picture response to create an environment in which older adults are encouraged to thrive as they age. In the subtheme ‘Solutions go beyond health care’, participants critiqued the overemphasis on medical responses during the pandemic. In the subtheme ‘Preparedness through prevention’, participants call for a shift toward upstream, preventive approaches. The subtheme ‘Building partnerships to support older adults’ stresses collaboration across sectors and between formal and informal networks.

#### Solutions go beyond health care

Across all focus groups, participants expressed strong criticism of the predominantly medical approach that shaped the pandemic response. While acute interventions and infection control were seen as necessary in the early phases, there was widespread concern that this narrow focus came at the expense of broader strategies essential to older adults’ overall well-being.

This critique was voiced most explicitly by policymakers, who highlighted the neglect of public health, social infrastructure, and preventive approaches. Rather than focusing solely on protecting against infection, they advocated for a more holistic perspective that considers housing, income, education, and social cohesion as equally vital components of well-being. Although they acknowledged that prioritising health care was understandable in the acute phase, they warned against allowing this singular focus to persist beyond the immediate emergency.

*“Health care is only one domain of people’s daily lives. There is also housing*,* income*,* education*,* and social connections to consider. And during crisis*,* the emphasis was laid on one domain*,* which was very important but should be scaled down as soon as possible and put on an equal footing with the other factors that determine people’s lives.”* Policymaker, focus group 4.

A similar call came from representatives of older adults, who emphasised the need for a cultural shift in how older adults themselves approach ageing. Rather than relying solely on formal systems, they emphasised that older adults should be encouraged to take an active role in shaping how they age:

*“It also has to come from the elderly themselves: preparing yourself for the future*,* think about where you live*,* whether you have a social network*,* what facilities are nearby*,* what do you do for other people*,* […] and actually start practicing that*,* and not just wait around and look at the government. That’s also a part of it.”* Older adult, focus group 1.

Community care professionals echoed these concerns and framed them through their professional lens. They reflected critically on what they saw as a widespread overdependence on formal care services. One participant described this societal orientation as a kind of “addiction to care.” According to this view, reliance on institutional care has inadvertently discouraged personal responsibility and community-based solutions, creating expectations that the system cannot sustainably meet:

“*Personally*,* I’ve become more and more convinced that we live in a society that has become addicted to health care. And with this*,* a whole group of people has been lulled into thinking: you don’t have to do anything yourself*,* we will take care of you [refers to care system]. And we now know that that is not going to work.*.” Policymaker, focus group 4.

#### Preparedness through prevention

Participants pointed to a longstanding neglect of preventive care for older adults in Dutch health policy. The pandemic was widely seen as a wake-up call that exposed this gap and underscored the need to invest in preventive strategies across the life course. Policymakers reflected critically on the disproportionate attention given to youth health, creating missed opportunities to enable older adults to be resilient and independent while ageing:

*“We do have preventive care for youth*,* but we haven’t focussed on prevention for adults and older people for years. All attention has gone to young people.*” Policymaker, focus group 4.

Across focus groups, participants described prevention not as a standalone service, but as something that should be embedded into the daily routines of older adults. Community care professionals emphasised the importance of accessible, low-threshold activities, such as physical exercise and cultural engagement. Yet, they described these options as undervalued and inconsistently available:

*“Anyway*,* it is my ambition to do much more for elderly people with exercise and culture*,* to do more with that early on. In a way that it is sustainable*,* because at the moment this is not true at all.”* Policymaker, focus group 5.

Importantly, participants across focus groups noted that the responsibility for preparedness and prevention does not lie with institutions alone. Echoing earlier calls for personal agency, they described older adults as active agents in shaping their futures, highlighting the importance of early planning, maintaining social networks, and seeking support proactively rather than reactively while ageing:

*“But this has to come from the elderly themselves to right*,* to prepare for the future*,* thinking about where you live*,* whther you have a network*,* are there facilities nearby*,* what do you do for others*,* right? And what might you ask from others. And don’t just wait for something to happen for which you need help.”* Older adult, focus group 1.

#### Building partnerships to support older adults

The pandemic was widely described as a catalyst that broke down professional silos and accelerated partnerships between health care providers, municipalities, social organisations, and local community actors. According to policymakers and community care professionals, crises created a unique momentum for collaboration, turning urgency into action. The call was not just for collaboration in times of crisis, but for an ongoing, integrated approach to ageing well.

*“The moment a crisis hits*,* like during COVID or now with the refugees*,* we try not to work in isolation but to pool our efforts. That gives us something concrete to act on in the case of a crisis*,* but we have been working for some time to get that collaboration going [refers to period before the pandemic].“* Policymaker, focus group 5.

Informal caregivers and community care professionals pointed to trusted relationships, such as those with home care providers, churches, or neighbourhood groups, as valuable foundations for collaboration, underscoring the importance of sustained community investment and infrastructure well before a crisis occurs. They emphasised the importance of identifying, preserving, and scaling these local efforts to strengthen preparedness for future crises:

*“Mmm there’ve been so many initiatives*,* but in the rush to do everything well*,* I think there’s been too little attention to the real gems. Those are the good ideas that you can also use again if something else happens.”* Informal caregiver, focus group 6.

Local networks were not only seen as complementary to professional services but as essential components of crisis response and long-term care strategies. Several participants emphasised that residents themselves, not just professionals, play a key role in maintaining resilient systems of support:

*“Well*,* prevention is better than curing*,* I think. If you already have strong social networks in your neighbourhood and if there is already a solid cooperation between professionals and residents*,* […] then in cases when health care falls short*,* we [refers to local initiative to provide support to people in the village] can take over and work together very nicely.”* Community care professional, focus group 1.

### Theme 3: strengthening personal pathways to well-being

Theme 3 is individual and community-centred, illustrating how structural and systems-level strategies to support well-being translate into everyday care for older adults. The subtheme ‘Fostering capacity and resilience’ highlights the importance of empowering older adults to maintain autonomy and resilience, thereby preventing unnecessary dependence, by creating environments where they can remain active agents in their own lives. The subtheme ‘Distinct ways to stay in touch with older adults’ emphasises practical and creative strategies for maintaining meaningful contact with older adults when resources are constrained. In the subtheme ‘Social inclusion as a pathway to well-being’, inclusion and connection are framed as a protective factor for older adults’ well-being.

#### Foster capacity and resilience

Across focus groups, participants reflected on how the pandemic intensified the tension between older adults’ increasing vulnerability and the need to support their autonomy. While the intention to protect was widely acknowledged, community care professionals pointed out that some care practices adopted during the crisis inadvertently undermined older adults’ sense of agency. They expressed concern that well-meaning caregivers may unintentionally “take over” tasks that older adults were still capable of doing themselves. This could discourage initiative and increase dependency:

*“As a caregiver*,* you can also keep someone dependent unintentionally*,* just because you always do things for them. Then the other person will think*,* “well*,* I probably can’t do it*,*” or they stop taking the initiative. And if you take that away*,* then you give them the chance to grow and take matters into own hands again.”* Policymaker, focus group 3.

This concern was echoed by informal caregivers and representatives of older adults, who described how the prolonged nature of the pandemic, and ageing itself, contributed to shifts in behaviour, with older individuals becoming more hesitant or reliant on others for tasks they previously handled independently:

*“Well*,* times have changed due to COVID*,* and that they [refers to other participants in the focus group] indicate that they ask for help more now*,* in some way we do too. We also involve the children more than we did before that time [refers to period before the pandemic]. Because we used to always say “we can manage*,*” but maybe that is also because we are getting older.”* Older adult, focus group 6.

To counter this trend, participants emphasised the need to foster resilience and strengthen older adults’ capacity to manage independently. Community care professionals suggested practical strategies, such as improving digital literacy to help older adults maintain social connections and access services. Representativeness of older adults confirmed the value of these skills, noting that a lack of digital competence made them more isolated during the pandemic. Alongside digital inclusion, participants advocated for designing community-based support systems that actively engage older adults and build on their strengths:

*“You should realise that it is really important to encourage people to build their own resilience and to give them the tools for it. […] And then you can handle not only COVID*,* but really anything life might throw at you.”* Policymaker, focus group.

#### Distinct ways to stay in touch with older adults

A shared observation across focus groups was that many older adults withdrew socially during the pandemic and struggled to re-engage even after restrictions were lifted. This was seen not only among frail individuals but also among previously active older adults, such as volunteers or community members involved in local initiatives. Participants described how the disruption of daily routines and social roles created a psychological barrier that made returning to pre-pandemic levels of participation especially difficult. As a representative of older adults explained, the sudden and prolonged interruption caused confusion and uncertainty, particularly for those not mentally prepared for such an abrupt change:

*“I think that*,* in general*,* people didn’t know that to expect. As far as I am concerned*,* that also has to do with*,* well*,* suddenly it was COVID*,* everything changed for many people*,* and they weren’t really prepared. At that point*,* everything came to a halt*,* and they were left thinking “so*,* what’s going to happen?”* Older adult, focus group 1.

Community care professionals pointed to the development of new, less socially active routines during lockdowns, which were hard to break even once restrictions eased:

*“You were used to going somewhere*,* and that stopped. Then you get used to a new rhythm*,* it might not be all that pleasant*,* but it takes over*,* and now it takes quite an effort to return to your old ways.“* Policymaker, focus group 3.

Rather than expecting older adults to take initiative themselves, participants emphasised the need for active and sustained outreach. Community care professionals explained how they used weekly phone calls and in-person check-ins to maintain contact with older adults and identify needs that might otherwise go unspoken. One representative of older adults stressed that older adults often require personal encouragement to participate, as self-initiative was not always present, particularly in vulnerable groups:

*“In order to get these people* [refers to older adults] *to participate*,* you really have to take them by the hand and say*,* “come on*,* join us*,* it’s fun”. But with that kind of thing*,* you have to…*,* they don’t show that much initiative*,* I don’t think.”* Older adult, focus group 1.

Another observation of participants was the psychological impact of uncertainty and inconsistent public health messaging. Especially informal caregivers stressed that incoherent and delayed communication contributed to confusion, fear, and disengagement. The focus on restrictions and risk led to a climate of fear, while little attention was given to empowering older adults with strategies to maintain their mental and social strength. Across focus groups, participants suggested that clear, consistent, and targeted communication could have reduced this anxiety:

*“Well*,* all this time it’s been based on fear*,* restrictions*,* restrictions*,* and that there was actually very little*,* you don’t see it now either*,* well*,* that things are allowed again*,* but that you still can strengthen your mental and physical resilience and there’s no propaganda for it*,* so to speak. Whereas I think*,* yeah*,* this is incredibly important*,* especially strengthening people’s mental resilience.”* Policymaker, focus group 5.

#### Social inclusion as a pathway to well-being

Participants highlighted loneliness and social isolation as major challenges faced by older adults during the pandemic. A community care professional described how preventive home visits revealed that many older adults had significantly deprived themselves of social and sensory stimulation during the past years, even if they did not explicitly label their experience as loneliness:

*“So*,* then you also see that side where older people have denied themselves a lot. I can’t say that they say*,* ‘we have been lonely*,*’ but they are*,* the fact that they have been deprived of contact and haven’t left the house*,* we do get strong signals to that effect.”* Policymaker, focus group 5.

Across focus groups, participants noted that older adults’ ability to maintain or adapt social activities depended largely on individual resilience and existing social networks. They emphasised the importance of keeping the social environment alert and attentive, encouraging people to remember and look out for each other, especially vulnerable older adults:

*“And that*,* again*,* has to do with the social environment*,* whether you are able to find other ways to continue your activities*,* whether you have the resilience and the flexibility so to say to do that. And whether you are in a situation where that is possible*,* that also makes a big difference.”* Policymaker, focus group 3.

## Discussion

This focus group study aimed to examine how to support older adults’ well-being in the context of constrained resources within health and social care systems, drawing on insights from the COVID-19 pandemic. Despite participants’ varied backgrounds, including community care professionals, informal caregivers, older adult representatives, and public health experts, three key themes consistently emerged across all groups and were discussed from different perspectives. Firstly, there was a shared call to focus on person-centred approaches that accommodate the diverse realities of older adults, despite limited resources. Secondly, participants emphasised the need for strategies that go beyond medical care, focusing on prevention, planning, and collaboration across sectors. Thirdly, participants stressed the importance of empowering older adults to maintain autonomy, resilience, and social connection through proactive outreach and community-based support. Together, these findings underscore the need for adaptable, inclusive, and forward-looking approaches to ageing in times of scarcity.

The findings of our study align with the public health framework for healthy ageing, which emphasises that health systems should move beyond disease curation and toward fostering functional ability and intrinsic capacity across the life course [5]. However, in the Netherlands, as elsewhere, support for older adults during the COVID-19 pandemic remained predominantly focused on medical care, with policy responses centred on infection control [33, 34]. As a result, non-acute care was often postponed or cancelled, and critical social determinants of older adults’ well-being were largely neglected, leading to adverse effects across all domains of their well-being [35]. This tendency toward infectious disease control and medicalisation is increasingly recognised in post-pandemic discussions on health system reform [35, 36]. Our study suggests that the principles of supporting healthy ageing outlined in the framework remain strongly endorsed by community-care stakeholders and older adults, even in the context of public health crisis. This underscores the need to rethink care structures for older adults in times of crisis, not only to address acute medical needs, but also to account for the broader social, environmental, and behavioural factors that profoundly influence the well-being of older adults [33, 34].

In line with other studies, our findings show that rigid, top-down approaches during the pandemic struggled to accommodate the specific needs of older adults, thereby constraining the delivery of person-centred care [37, 38]. With this, the COVID-19 pandemic exposed an enduring tension between centrally mandated protocols and the need for local adaptation in health and social care delivery [15, 20, 34]. Importantly, this tension is not unique to crisis contexts but reflects a broader, ongoing challenge in healthcare: how to balance person-centred, community-responsive care with the equity, consistency, and coordination afforded by standardised systems [39]. Specific elements hindering the provision of person-centred care were prolonged reductions in care capacity, losses of routine-person contact and a lack of recognition that older adults are a heterogenous group with variating care needs, which are also known barriers to person-centred care in non-crisis situations [40, 41]. This illustrates that person-centred care is not merely a matter of professional attitude or intent, but is fundamentally contingent on system design, including governance structures, resource allocation, and the degree of integration across sectors.

Furthermore, the findings stress that maintaining consistent care practices and supporting the daily routines of older adults are crucial to sustaining their well-being and independence. These insights highlight the importance of implementing reablement strategies as part of broader approaches to support healthy ageing. In the Dutch context, reablement refers to efforts to enhance individuals’ autonomy and self-reliance and independence in everyday life with a particular focus on social participation [42, 43]. Our study reinforces this broader understanding of reablement by showing the importance of in-person contact and maintain social routines for the well-being of older adults despite public health crisis. In the context of constrained health and social care systems, prioritising this more holistic operationalisation of reablement is essential, shifting the focus away from task-oriented or efficiency-driven health and social care models toward approaches that actively support autonomy, resilience, and social inclusion in later life [43].

Finally, participants highlighted the vital role that older adults themselves play in preparing for ageing. This involves proactive planning across physical, financial, social, psychological, and environmental domains to adapt to age-related changes [43–46]. Moreover, they stressed that preparation for ageing is a public health priority that expands beyond personal concerns. This is in line with recent evidence, showing that pre-existing healthy lifestyle behaviours and social networks significantly buffered against negative mental health outcomes during crises like the COVID-19 pandemic [46, 47]. This highlights that building older adults’ resilience before a crisis occurs is beneficial for individual well-being and an essential pillar of pandemic preparedness.

A key strength of this study lies in its inclusion of diverse stakeholder perspectives captured through focused, small-group discussions. This multi-actor approach enriched the findings by highlighting how systemic and organisational challenges interact with individual experiences across care contexts. Moreover, the use of abductive reasoning and thematic analysis enabled a nuanced, theoretically grounded interpretation responsive to the complex issues surrounding older adult well-being in the disruptive context of the COVID-19 pandemic.

Our study also has limitations. A first limitation concerns the heterogeneity of the sample. Although diversity was sought, policymakers are overrepresented in the study population. This likely to be due to recruitment via the FAITH research network, potentially privileging policy-oriented over practice-based perspectives. In addition, the online format may have affected participation of individuals with lower digital skills, particularly for people of older age, who are known to have lower levels of digital confidence and access [23]. However, increased digital uptake during the COVID-19 pandemic may have reduced this impact. Another limitation was that the online format may have affected participation of those with lower digital skills, creating technical barriers that affected their ability to contribute fully. This was mitigated through technical support and active moderation to ensure inclusion by the assistant and moderator. Furthermore, the mixed professional background in two focus groups may have introduced power asymmetries that influenced group interaction and openness of discussion. We believe that the use of Mural supported equal input to mitigate this. A limitation regarding data analysis is that we did not explicitly attend to the interactional dynamics within the focus groups. Our analysis primarily focused on the content of participants’ contributions rather than how meanings were jointly produced through group interaction. Finally, the findings are situated within the Dutch care context and the specific conditions of the COVID-19 pandemic, which may limit their transferability to other health systems or non-crisis settings.

In conclusion, although the COVID-19 pandemic has passed, many of the challenges in caring for older adults identified in this study persist beyond the pandemic context. Our findings highlight that promoting the well-being of older adults requires continuous commitment to preventive, context-sensitive approaches, even under resource constraints. This entails embedding flexibility within national frameworks to adapt to local contexts and fostering cross-sector collaboration to deliver truly person-centred care. Equally important is creating environments that empower older adults to actively prepare for ageing. As health systems confront ongoing and future challenges, integrating these insights into policy and practice is vital for building inclusive, responsive, and resilient care systems for ageing populations.

## Supplementary Information

Supplementary Material 1.

## Data Availability

The datasets used and/or analysed during the current study are available from the corresponding author on reasonable request.
