# Supplementary material for: Community stakeholders’ perspectives on supporting the well-being of older adults: a focus group study on lessons learned during the COVID-19 pandemic
Source: BMC Public Health. 2026 May 22;26:2147. doi: 10.1186/s12889-026-27862-8 (PMC13371319; doi:10.1186/s12889-026-27862-8)
Supplement: Supplementary file 1 — Supplementary Material 1. [file 12889_2026_27862_MOESM1_ESM.docx]

**Supplementary material 1: topic list**

**Introduction**

*Plenary, facilitator:*

- Welcome
- Explain purpose of study
- Give practical information: use of Mural, duration, anonymous reporting, audio recording.
- Explain roles and guidelines: the facilitator will monitor time and ensure that everyone can contribute; confidentiality, respect, and other group agreements apply.

*Plenary, everybody:*

- Brief introductions

**Part 1: focus changes during the COVID-19 period**

*Facilitator asks participants to go to Mural and fill in first part.*

*Then, the outcomes are discussed plenary. Guiding questions are:*

- What were observed changes among clients/target group?
- What were changes in working methods during the pandemic?
- How did participants adapt to altered working methods?
- What were changes in the client–care provider relationship?
- What were changes in multidisciplinary collaboration?

**Part 2: risk factors and coping strategies**

*Facilitator asks participants to go to Mural and fill in first part.*

*Then, the outcomes are discussed plenary. Guiding questions are:*

- Which risk factors contributed to increased frailty within clients/target group? (e.g., individual factors, environmental factors)
- Which protective factors helped clients/target group remain less frail during the pandemic? (e.g., individual factors, environmental factors)
- What coping mechanisms were observed among clients/target group?

**Part 3: recommendations for the future**

*Facilitator asks participants to go to Mural and fill in first part.*

*Then, the outcomes are discussed plenary. Guiding questions are:*

- How can adverse effects be prevented?
- What does *not* work?
- How can participants’ own organisations contribute?
- How can the national government contribute?
- How can the local municipality contribute?

**Closing**

*Plenary discussion, final question:*

- Is there anything participants would like to add that has not yet been discussed?

*Plenary, facilitator:*

- Thanking participants
- Explain next steps: data collection, data analysis, data publication

**General probes:**

- What do you mean by …?
- Could you tell me more about that?
- Could you give an example of what you mean?
- What makes you feel that way?
- I’m not sure I fully understand. Could you explain it?
- Can you explain why that is important to you?
